# Divergent Temporal Response of Abundant and Rare Bacterial Communities to Transient *Escherichia coli* O157:H7 Invasion

**DOI:** 10.3389/fmicb.2021.665380

**Published:** 2021-06-07

**Authors:** Nan Zhang, Chunling Liang, Xiangjun Liu, Zhiyuan Yao, David Z. Zhu, Shicong Du, Huajun Zhang

**Affiliations:** ^1^School of Civil and Environmental Engineering, Ningbo University, Ningbo, China; ^2^Institute of Ocean Engineering, Ningbo University, Ningbo, China; ^3^Zhejiang Provincial Key Laboratory of Agricultural Resources and Environment, Hangzhou, China; ^4^School of Marine Sciences, Ningbo University, Ningbo, China

**Keywords:** *Escherichia coli* O157:H7, invasion, microbial assembly, niche, rare biosphere

## Abstract

The release of *Escherichia coli* (*E. coli*) O157:H7 has been widely found in various environments, but little is known about the probable influence of the transient *E. coli* O157:H7 invasion on the native microbial community. Here, we investigated the temporal response of two bacterial biospheres (abundant and rare) of two marsh sediments against *E. coli* O157:H7 during a 60-day incubation. The diversity of both biospheres showed no evident response to O157:H7 invasion. Temporal factor exhibited greater effects on bacterial variation than O157:H7 invasion. We found that O157:H7 invasion led to an increase in the niche breadth of the bacterial community while decreasing the efficiency of bacterial interaction of the abundant taxa. Moreover, the rare biosphere exhibited enhanced stability against O157:H7 invasion compared with the abundant biosphere, acting as the backbone in resisting external disturbance. Furthermore, each subcommunity assembly showed different randomness levels. The stochastic events were relatively more important in constraining the abundant taxa assembly after invasion. Collectively, *E. coli* O157:H7 exhibited diverse tangible impact on both biospheres, which unearthed differential responses of abundant and rare biosphere against transient microbial invasion.

## Introduction

The rapid spread of microorganisms into various ecological systems is known to impact the function and structural make-up of the native microbial communities (Li et al., 2019; Thakur et al., 2019). Thus, current researches in the fields of microbial ecology and invasion biology have been focused on the identification of novel microbial invasion along with the assessment of their microecological impact (Wei et al., 2018; Li et al., 2019; Braga et al., 2020).

Extensive studies have been conducted on invasion by phytopathogens since they gradually displace the resident communities, resulting in significant economic losses in agriculture (Levine et al., 2004; Robin et al., 2011; Wei et al., 2015, 2018). Furthermore, zoonotic pathogens are also known to invade the natural microbial communities (Van Elsas et al., 2011; Liang et al., 2019). The release of livestock feces into the soil might result in potential invasion by approximately 10^9^ *Escherichia coli* (*E. coli*) cells/g feces. Different from the phytopathogenic invaders, zoonotic pathogens are highly abundant in the initial phase. However, they seldom transform into autochthonous species (Ishii et al., 2006; Mallon et al., 2018). This observation raises the question whether the transient invaders, such as zoonotic pathogens, could induce a change in the resident micro-community. Recent studies showed that transient invaders altered the composition and niche structure in synthetically assembled communities of microbes (Mallon et al., 2018; Li et al., 2019; Xing et al., 2020). Since synthetic communities are simplified than their natural counterparts, more knowledge is required regarding the alteration in natural microbial community.

Bacterial communities are known to be comprised of ubiquitously abundant species as well as several rare species (Pedros-Alio, 2012). The abundant species account for a significant proportion of bacterial biomass and biogeochemical cycling, while the rare species are critical for the ecosystem stability and functions (Pedros-Alio, 2012; Jia et al., 2018; Du et al., 2020). Recent investigations have revealed how abundant and rare bacterial subcommunities respond distinctively to external disturbances, such as diatom blooms (Zhang et al., 2020), inorganic carbon stress (Shu et al., 2018), organic pollution (Jiao et al., 2017), etc. However, there is limited information on the impact of microbial invaders on rare biosphere of sediment. Thus, it is imperative to explore the response of both abundant and rare species against microbial invaders for an improved understanding of the microecological role of transient invaders in native microbial communities.

Interspecies interactions have an indispensable position in structuring the development of bacterial communities (Faust and Raes, 2012; Gao et al., 2019). The overall influence of unsuccessful invaders on the co-occurrence pattern and central species remains unclear. Additionally, the ecological processes underlying the patterns observed in relation to species abundance across time and space are vital in microbial ecology (Nemergut et al., 2013; Dini-Andreote et al., 2015). Both the deterministic and stochastic processes concurrently act for the regulation of the microbial community assembly (Nemergut et al., 2013; Zhou and Ning, 2017). However, the degree to which differences in the balance between these two processes is driven by the microbial invader needs to be investigated. Previous studies were mostly focused on a single time point after microbial invasion and did not explore the variation in microbial population with time (Mallon et al., 2018; Li et al., 2019; Xing et al., 2020). Thus, a set of successional systems post-microbial invasion would be ideal to unravel the effect of invaders on the ecological processes governing the microbial assembly.

The release of *E. coli* O157:H7, a common cause of foodborne illness (Van Elsas et al., 2011), into natural environment facilitates its interaction with the indigenous microorganisms (Yao et al., 2014, 2019b; Mallon et al., 2018). Our research group has previously found that *E. coli* O157:H7 persists in marsh sediment for 36 to 53 days (Liang et al., 2019). However, only little relevant details are available regarding the influence of intruding *E. coli* O157:H7 on the marsh bacterial community, which harbors high biodiversity and important ecosystem functions (Yao et al., 2019a). Here, we examined the effects of invading *E. coli* O157:H7 on both abundant and rare biosphere of two distinct marsh sediments by investigating the variation in diversity, composition, co-occurrence pattern, and assembly processes with time. The alterations in these parameters would correspond to identical mechanisms unraveling how different communities respond to invasion.

## Materials and Methods

### Sediment Description

The surface marsh (5–20 cm) sediments (B and P) which was covered with distinct vegetation (bare and *Phragmites australis*, respectively) were collected in the estuarine marsh (30°21′56″N, 121°12′39″E) in south Hangzhou Bay, China (Supplementary Figure 1; Yao et al., 2019a). The B sediment contained 3.4 ± 0.4 g/kg organic carbon, 68.5 ± 8.8 g/kg ammonia nitrogen, 0.2 ± 0g/kg total nitrogen and had a pH of 6.7 ± 0.2. The P sediment contained 6.8 ± 0.6 g/kg organic carbon, 134.2 ± 9.8 g/kg ammonia nitrogen, 0.6 ± 0.1 g/kg total nitrogen and had a pH of 8.0 ± 0.1. No indigenous *E. coli* O157:H7 was detected in both sediments.

### Experimental Setup and Procedures

*Escherichia coli* O157:H7 (non-pathogenic *E. coli* O157:H7 strain 3704 Tn5 luxCDAEB) was introduced into the sediment at 10^7^ CFU/g, and the water content was adjusted to 100% water-holding capacity. This inoculation density represented a classical invading *E. coli* in nature, which was in agreement with previous studies (Fukushima and Seki, 2004; Yao et al., 2013, 2019b; Liang et al., 2019), providing a reliable template to examine the effects of *E. coli* O157 intrusion. Sediment samples inoculated with equivalent amount of sterile water served as non-invading controls. The inoculated samples were incubated in a dark environment at constant temperature (25 ± 1°C) and constantly maintained by adding sterile deionized water every 2 days to make up for the moisture loss during incubation. After 36 and 48 days, there was no culturable *E. coli* O157:H7 detected in B and P sediment (Liang et al., 2019). To discover the existence of lag time in indigenous bacterial communities in response to the invasion, we sampled 12 days after no culturable *E. coli* O157:H7 was detected in each sediment. Correspondingly, the sediment B was destructively sampled on days 0, 5, 20, 36, and 48. The sediment P was destructively sampled on days 0, 5, 20, 36, 48, and 60. Each sampling, we stored approximately 10 g of sediment from individual treatment at a temperature of −80°C for DNA extraction.

### DNA Extraction, PCR, and Sequencing

The DNA isolation kit PowerSoil (MOBIO, United States) was employed for sediment DNA extraction according to the manufacturer guidelines. A NanoDrop spectrophotometer (NanoDrop Technologies Inc., United States) established the DNA concentration and quality. The V4 region of the bacterial 16S rRNA gene was amplified using the following primers: 515F (5′-GTGYCAGCMGCCGCGGTAA-3′) and 806R (5′-GGA CTACNVGGGTWTCTAAT-3′) (Parada et al., 2016). The PCR reaction was carried out in a reaction mixture of 20 μl under the following thermo-cycling operations: 4°C for 3 min, 30 cycles of 95°C for 30 s, 50°C for 30 s, and 72°C for 45 s. This was followed by extension at 72°C for 10 min. We pooled equimolar amounts of the purified products of PCR of individual sample and sequenced them using the Illumina MiSeq platform (Illumina, Inc., San Diego, CA, United States) having a paired-end (2 bp × 300 bp) approach. The NCBI accession number of the raw sequencing data was PRJNA669260.

### Sequence Data Processing

Raw sequencing data were processed using QIIME 2^1^, followed by denoising and filtration using plug-in DADA2 database. Subsequently, the Amplicon Sequence Variants (ASVs) was identified by a naïve Bayes classifier that was trained with the plug-in feature-classifier employing the 16S rRNA gene database having a 99% similarity of the SILVA 132. Post-quality trimming, 1,398,000 reads of high-quality remained in the dataset, yielding 16,595 ASVs. The random rarefication was carried out at the similar sequence depth (at 23,300 sequences in a single sample) to make correction for varying sampling efforts. We used this ASV table for all further analyses. Abundant and rare ASVs were categorized based on relative abundances > 0.01% per sample and < 0.005% across all samples, respectively. R v3.5.3^2^ was used to conduct both alpha (α) and beta (β) diversity analyses. We estimated the Shannon index and made use of the Weighted UniFrac distance for β-diversity. The Bray–Curtis dissimilarity of β-diversity was used for time-lag regression. Niche breadth was calculated using “spaa” package in R v3.5.3.

### Co-occurrence Network and Community Assembly Analysis

To improve statistical confidence, ASVs present in at least 20% of samples were retained for the construction of networks. Subsequently, all possible pairwise Spearman’s rank correlations (ρ) between those ASVs were calculated. Only robust (ρ > 0.6 or ρ < −0.6) and statistically significant (*p* < 0.01) correlations were incorporated into network analyses. Module analysis and topology results were made with Gephi interactive platform. Modules were identified by the Louvain algorithm. Then, the topology of Erdös–Réyni random networks was calculated with same node and edge. Finally, the network visualization of dominant taxa was plotted by chord diagram.

### Ecological Stochasticity Estimation and Determinism in Community Assembly Analysis

To understand bacterial community assembly processes, we calculated the estimated normalized stochasticity ratio (NST) by adopting the null model to ascertain the tochasticity value in community assembly to assess the relative influence of ecological activities. If NST < 50%, it represented a more deterministic assembly while NST > 50% denoted a more stochastic assembly. The “tNST” function was used to conduct this analysis with the following parameters: “dist method” of “bray,” “abundance weighted” of “TRUE,” and “rand” of “1,000” in the “NST” package.

### Statistical Analysis

Significant difference in the α-diversity and niche breadth were tested by making use of ANOVA (one-way analysis of variance) and significant difference of bacterial composition was tested by Mann-Whitney U test. All test analysis was made by SPSS statistical software (SPSS, United States). We used the Bray–Curtis dissimilarity to conduct permutational multivariate analysis of variance by using the ADONIS function to calculate the differences in community compositions among samples. Additionally, the Weighted UniFrac distances were used to perform a principal coordinates analysis (PCoA) to pinpoint the similarity among various bacterial groups. R statistical software was employed for the analysis unless indicated otherwise.

## Results

### Generalized Patterns of α-Diversity and Bacterial Composition

**FIGURE 1:**
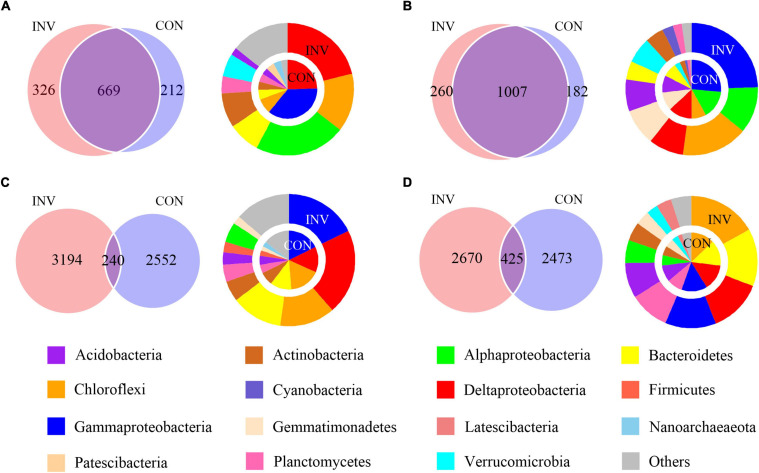
Venn diagram representing the numbers of shared and amplicon sequence of variants (ASVs) among the CON and the INV group. The average relative abundances of the phyla (relative abundance above 2%) in each special category are shown in the pie chart. CON, the control group; INV, the invasion group. **(A)** Abundant taxa of the B sediment **(B)** Abundant taxa of the P sediment **(C)** Rare taxa of the B sediment **(D)** Rare taxa of the P sediment.

We classified 2,428 ASVs (14.63%), encompassing 58.31% of all sequences, as abundant taxa and 11,459 ASVs (69.05%), encompassing 22.75% of all sequences, as rare taxa. The rare subcommunity showed higher level of α-diversity compared with the abundant (*p* < 0.05), indicating a more diverse taxonomy in the rare biosphere (Supplementary Figure 2). Additionally, we found no considerable difference between the invaded group (INV) and the non-invaded control group (CON) for both sediments (Supplementary Table 1). Some phyla, including Chloroflexi and Proteobacteria, were either found profusely in every ecosystem, whereas some phyla, such as Latescibacteria, Bacteroidetes, Nitrospira, and Planctomycetes were always rare (Supplementary Figure 3). In addition, the abundant and rare taxa in B and P sediments show no significant difference after invasion at the phylum level (Supplementary Figure 4). We hypothesized that the invading O157:H7 did not have a direct impact on the bacterial composition regarding dominant taxa distribution and might be reflected in a lower classification level. The Venn diagram revealed considerable overlap between the INV and CON groups for the majority of abundant ASVs (Figures 1A,B), whereas the rare taxa included most of the treatment-specific ASVs (Figures 1C,D). For the abundant taxa in the B and P sediments, more ASVs belonging to Verrucomicrobia and Chloroflexi were found in the INV group (Figures 1A,B). Additionally, the dominant role of γ-proteobacteria in the CON group was replaced by other phylum in B sediment (Figure 1A). However, we did not observe a consistent change between the B and P sediments in the rare taxa (Figures 1C,D).

### Successions of Abundant and Rare Taxa After O157:H7 Invasion

**FIGURE 2:**
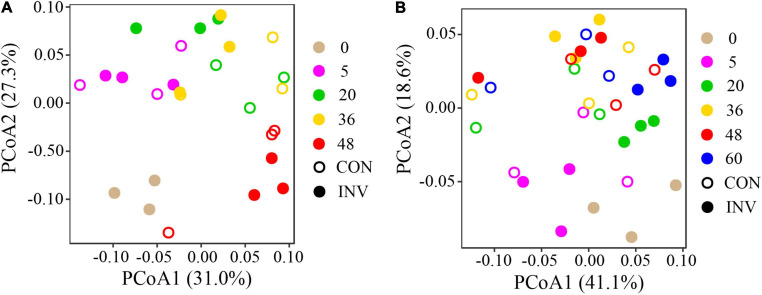
Principal coordinate analysis (PCoA) of the bacterial communities based on the Weighted Unifrac distance. **(A)** B sediment **(B)** P sediment. CON, the control group; INV, the invasion group.

**FIGURE 3:**
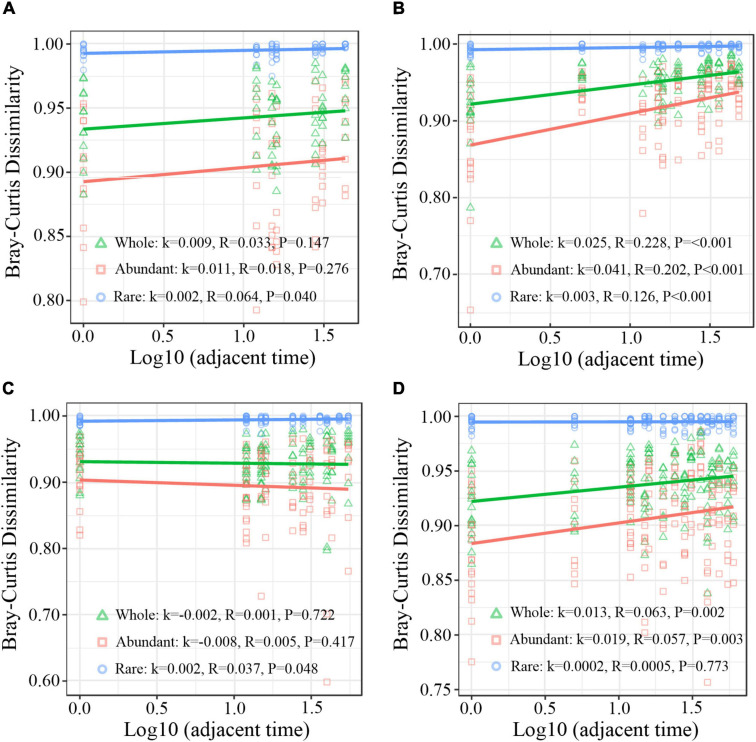
Time-lag regression analysis of the relationship among sampling time and Bray–Curtis dissimilarity. **(A)** B sediment of the CON group **(B)** B sediment of the INV group **(C)** P sediment of the CON group **(D)** P sediment of the INV group. *k*, the regression slope; Whole, all bacterial community; Rare, rare taxa; Abundant, abundant taxa; CON, the control group; INV, the invasion group.

**FIGURE 4:**
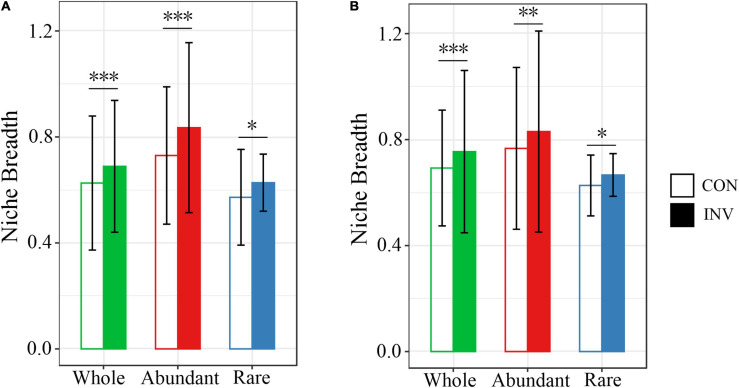
Barplots showing the mean niche breadth for the whole, abundant, and rare bacterial communities of the B **(A)** and P **(B)** sediments. The significance level after evaluation through the nonparametric Mann-Whitney *U* test (^∗^*p* < 0.05; ^∗∗^*p* = 0.001; ^∗∗∗^*p* < 0.001).

Principal coordinates analysis (PCoA) based on Weighted Unifrac distance was carried out to analyze the successional trajectories of whole bacterial structure (Figure 2). All samples were primarily clustered by time (ADONIS, R^2^ = 0.023, *p* = 0.013) rather than by invasion treatment (ADONIS, *R*^2^ = 0.016, *p* = 0.641), indicating that the temporal factor primarily regulated bacterial succession. Relatively apparent but not significant division between the INV and CON group was found in both abundant and rare sub communities in B and P sediments in the late stage (Supplementary Figure 5). The temporal turnover of abundant and rare subcommunities after O157:H7 invasion was further evaluated by the bacterial time–decay relationship (Figure 3). The temporal turnover of the INV group showed significant slopes and its rate was higher than the CON group, except the rare taxa of the P invasion group (Figure 3), suggesting that the bacterial communities of the INV group were undergoing a directional change. Also, β-diversity was attributed to richness of species instead of their replacement (turnover) based on the partition of β-diversity, which facilitated the transition in community constitution (Supplementary Figure 6). Finally, we estimated the niche breadths of community-level habitat to further explore the patterns of β-diversity (Figure 4). All samples had a greater value of niche breadth for the abundant taxa in comparison with the rare taxa (Figure 4). Moreover, the niche breadth values of the INV group were significantly higher than the corresponding CON group (*p* < 0.05) (Figure 4).

### Ecological Network Analysis on the Bacterial Community

**FIGURE 5:**
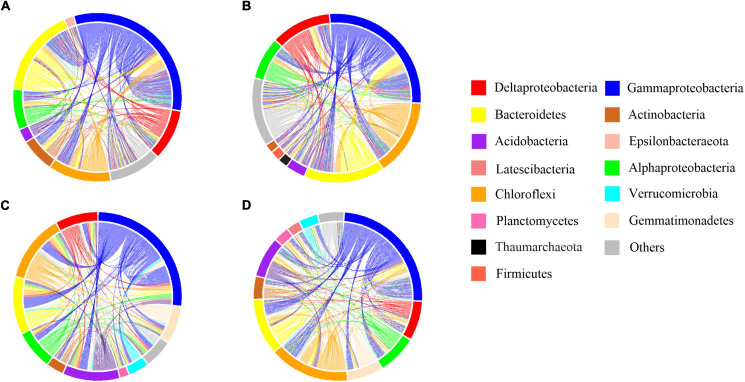
Co-occurrence patterns of abundant bacterial community. The profiles of co-occurrence links among dominant taxa. Connections are colored based on the most dominant taxon (relative abundance above 2%). **(A)** B sediment of the CON group **(B)** B sediment of the INV group **(C)** P sediment of the CON group **(D)** P sediment of the INV group.

**FIGURE 6:**
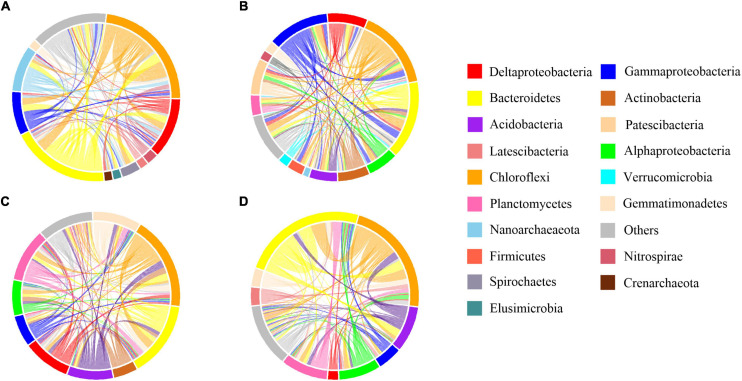
Co-occurrence patterns of rare bacterial community. The profiles of co-occurrence links among dominant taxa. Connections are colored based on the most dominant taxon (relative abundance above 2%). **(A)** B sediment of the CON group **(B)** B sediment of the INV group **(C)** P sediment of the CON group **(D)** P sediment of the INV group.

Network analyses were done to examine the impact of microbial invasion on bacterial co-occurrence (Figures 5, 6 and Supplementary Figure 7). The observed modularity and average clustering coefficient were all greater than those of their respective Erdös–Réyni random networks and small-world coefficient > 1, indicating that the network had “small-world” properties and modular structure (Table 1). The major topological characteristics of the networks were estimated to reveal the differences in co-occurrence pattern caused by O157:H7 invasion (Table 1). The high modularity values (>0.4) of all the networks implied the presence of modular architecture in all the samples (Barberán et al., 2012). Thus, the microbial invasion resulted in higher modularity for both subcommunities (Table 1). Additionally, both abundant and rare taxa showed slightly higher network densities in the CON group than in the INV group, indicating the presence of a more complex network in the CON group. The average clustering coefficient was lower in the abundant taxa of the INV group than the CON group, while no difference was found in the rare taxa (Table 1). Moreover, the average path length, which exhibited an opposite trend, was much higher in the INV group than the CON group, except the rare taxa.

**TABLE 1:** Topological properties of the real co-occurrence networks of whole, abundant, and rare communities and their associated random networks.

		Real networks							Random networks			
Group		Nodes^*a*^	Edges^*b*^	Modularity^*c*^	Average clustering coefficient^*d*^	Network diameter^*e*^	Average path length^*f*^	Network densities^*g*^	Modularity	Average clustering coefficient	Average path length	Small-world coefficient^*h*^
Whole	B-CON	577	2,253	0.953	0.978	4.00	1.02	0.014	0.284	0.013	3.32	246
	B-INV	818	3,418	0.953	0.862	16.0	4.86	0.010	0.259	0.011	3.40	54.8
	P-CON	1,031	4,979	0.921	0.823	38.0	13.4	0.009	0.233	0.010	3.31	20.4
	P-INV	1,196	4,582	0.967	0.875	60.0	18.2	0.006	0.267	0.006	3.71	29.7
Abundant	B-CON	312	585	0.946	0.969	3.00	1.02	0.012	0.447	0.011	4.39	378
	B-INV	373	622	0.968	0.959	6.00	1.10	0.009	0.489	0.007	4.88	610
	P-CON	504	1,041	0.937	0.959	8.00	1.43	0.008	0.428	0.007	4.52	432
	P-INV	685	1,404	0.957	0.784	17.0	4.99	0.006	0.443	0.005	4.81	151
Rare	B-CON	96.0	169	0.897	1.00	1.00	1.00	0.037	0.403	0.019	3.51	185
	B-INV	145	252	0.923	1.00	2.00	1.00	0.024	0.481	0.003	3.92	1,292
	P-CON	163	260	0.925	1.00	1.00	1.00	0.020	0.493	0.004	4.38	1,096
	P-INV	163	212	0.951	1.00	1.00	1.00	0.016	0.587	0.006	5.29	882

*^a^Number of ASVs with the correlation r > 0.6 or r < −0.6 and statistical significance (P-value < 0.01). ^b^Number of strong and significant correlations between nodes. ^c^Modularity > 0.4 suggests that the network has a modular structure. It indicates that there are nodes in the network that are more densely connected between each other than with the rest of the network and that their density is noticeably higher than the graph’s average. ^d^How nodes are embedded in their neighborhood, and the degree to which nodes tend to cluster together. ^e^The maximum distance between all possible pairs of nodes. ^f^The average number of steps along the shortest paths for all possible pairs of network nodes. ^g^The density of a graph is the ratio of the number of edges and the number of possible edges. ^h^Small-word coefficient > 1 indicates “small-world” properties, that is, high interconnectivity and high efficiency.*

### Ecological Assembly of Bacterial Communities

**FIGURE 7:**
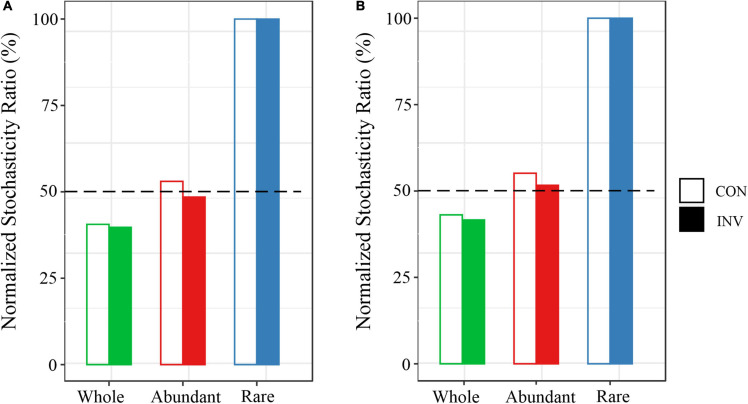
The ecological stochasticity in the assembly of the potentially pathogenic bacterial community evaluated based on the normalized stochasticity ratio (NST) based on the Bray–Curtis indexes.**(A)** normalized stochasticity ratio of B sediment **(B)** normalized stochasticity ratio of P sediment.

The neutral community model (NCM) estimates that a small ratio of the link between the frequency occurrence of ASVs and their relative abundance variations, ranging from 18.0–32.0% (Supplementary Figure 8). For any treatment, we consider a frequent occurrence of several bacterial taxa (below or above the neutral prediction) than predicted by the NCM based on their gross abundance in the metacommunity (Supplementary Figures 8, 9). Next, the null model-based NST was calculated based on the phylogenetic and taxonomic metrics to further investigate the community assembly mechanism shaping the bacterial community (Figure 7). Almost the estimated NST for both abundant and rare taxa > 50%. Additionally, the NST of rare taxa remained constant after microbial invasion, while that for the abundant taxa decreased.

## Discussion

The pollutants impact the soil bacterial community through diverse mechanisms, and the microorganisms often respond rapidly to these external changes (Jiao et al., 2016, 2017; Guo et al., 2020). *E. coli* O157:H7 invasion has been known to modify the structure of native indigenous community, resulting in enhanced microbial diversity as well as niche breadth (Yao et al., 2014; Mallon et al., 2018). Here, we had hypothesized that *E. coli* O157:H7 would alter the community diversity. However, we found no evidence regarding any significant influence of this microbial invader on the overall community diversity, structure, and composition of the marsh sediment bacterial communities (Figure 2, Supplementary Table 1, and Supplementary Figures 2, 4, 5). This observation contradicted the results of previous studies that involved synthetic communities (Mallon et al., 2018; Xing et al., 2020), which might be attributed to the increased biodiversity of native microbial communities compared with the synthetic communities (Xing et al., 2020). Also, it was possible that there were experimental effects which were not detected in this study. For example, perhaps the temporal scale of sampling (before invasion and then 5 days after the invasion) was too coarse and a more immediate sampling was needed to detect community response. We suspected that if communities were altered in the short term by *E. coli* O157:H7, the effects would be transient. In addition, there might be changes in the frequency of certain traits without an accompanying change in community membership. 16S rRNA genes sequencing would not detect such an effect, which require further metagenomic or functional analyses. Variation in bacterial β-diversity across samples can be ascribed to the sampling time. This indicated that post-microbial invasion, time was a considerably important factor in regulating the bacterial succession in the microcosms. However, the bacterial structure showed more obvious dispersion during the late stage in both sediments (Figure 2). We hypothesized that the impact of the microbial invader was not visible immediately and might have lasting effects on the bacterial community. This was consistent with Amor et al. (2020) who found that transient invaders could induce long-term shifts in complex microbial communities.

Our results showed the presence of common abundant taxa within the INV and the CON groups (Figures 1A,B), while a different distribution was observed for the rare taxa (Figures 1C,D). These results agreed with previous studies that abundant taxa were always distributed among various habitats (Du et al., 2020; Zhang et al., 2020). In addition, abundant taxa occupied broader niches than the rare ones (Figure 4), availing an array of resources supporting their survival. Furthermore, we observed a decrease in the γ-proteobacteria (class of *E. coli* O157:H7) in the abundant taxa of INV group (Figures 1A,B), due to niche competition because of the similar resource preferences with O157:H7, and this might trigger that the taxa better utilize or look out for niches that have not been exploited by O157:H7 (Mallon et al., 2018; Wu et al., 2021). Similar results have been communicated for *Pseudomonas* invasion in soil system, which reduced the relative abundance of resident bacteria which exploited the same resources (Fliessbach et al., 2009; Gómez et al., 2016). On the contrary, more abundant taxa affiliated to Chloroflexi and Verrucomicrobia, which are involved in the carbon cycle (Bergmann et al., 2011; Hug et al., 2013) were observed in the INV group (Figures 1A,B). Similarly, Wu et al. (2021) revealed that *E. coli* invasion resulted in enhanced relative abundance of Chloroflexi in residual biofilms of emergent aquatic plants. Furthermore, the change in community compositions post-invasion resulted in shifts in the niche structure as well as an increased niche breadth across all samples (Figure 4), which corroborates with the results of Mallon et al. (2018). This suggests that O157:H7 invasion facilitated the expansion of this population, which may ultimately lead to a functional change of sediment ecosystem. Further research should include functional genomes to gather deep information on this subject.

Species–time relationship (STR) has frequently been used to analyze microcosm communities in terms of microbial succession (Jiao et al., 2017; Zhang et al., 2020). The STR exponent provides a time-based estimation of the rate of occurrence of new taxa in a community, while a higher exponent indicates more newly introduced taxa (Preston, 1960). Here, the STR exponents of the microcosms (0.002 to 0.025) were lower than the slopes observed in bacterial succession in several natural habitats (Figure 3; Shade et al., 2013). This was attributed to the fact that the relatively closed environment induced by tightly controlled microcosms dramatically reduced the occurrence of new taxa introduced through dispersal. In the controlled experimental system, there was not any recruitment or input from an external species besides the invader (Mallon et al., 2018). Additionally, the STR exponent of the inorganic/organic pollutant-treated microcosms was higher than the microcosms with microbial invader in this study (Jiao et al., 2017). We speculate that the influence of inorganic/organic pollutants was obviously greater than the microbial invader. Also, the abiotic conditions of the microcosms are known to fluctuate due to pollutant degradation (Shade et al., 2013; Jiao et al., 2017), while the abiotic conditions remained constant in this study (data not shown). Despite this, we observed an increase in the STR slope of the total and abundant taxa post-microbial invasion, while the rare taxa remained constant in both B and P sediments (Figure 3). This indicated that the rare subcommunity have relatively greater stability under the influence of environmental pressure, consistent with other studies (Du et al., 2020). As compared with the abundant taxa, a higher α-diversity was observed in the rare taxa which has reported to be taxonomically and functionally diverse (Logares et al., 2014; Du et al., 2020; Zhang et al., 2020). Thus, the hypothesis put forth is that the rare taxa could easily achieve a more stable community after external disturbance. Hence, rare taxa are vital for supporting ecosystem stability against microbial invasion and could represent the hidden backbone of the microbial communities (Ziegler et al., 2018).

Network analysis is used to understand the impact of O157:H7 invasion on bacterial co-occurrence pattern. We observed a random arrangement modular framework in the bacterial networks (Table 1, Figures 5, 6, and Supplementary Figure 7), which were suitable to study the co-occurrence modes in the biological systems (Xue et al., 2018). Network topology reflected the variation in the pattern of bacterial interactions between treatments (Table 1). We observed a higher clustering coefficient than that reported for other natural ecosystems (Table 1), including polluted soil (Chao et al., 2016), original marsh sediment (Yao et al., 2019a), and seawater (Zhang et al., 2020), revealing stronger bacterial correlations in this microcosm compared with other ecological networks. This was indicative of niche filtering and cross-feeding relationships in such environment (Ma et al., 2020). Furthermore, the abundant taxa of the CON group exhibited an increased magnitudes of clustering coefficient, density and smaller mean path length than the corresponding INV group (Table 1). The shorter average path length could prompt the perturbations to reach the whole network and contribute to a more efficient system (Zhou et al., 2010; Faust and Raes, 2012; Jin et al., 2020). It is reasonable to infer that the abundant subcommunity of CON group was more complex and efficient since they could cope better with the external changes. Meanwhile, the clustering coefficient and the mean path length remained stable in the rare taxa post-invasion (Table 1), confirming the backbone role of rare taxa in resisting microbial invasion. The results of this study demonstrate that microbial invasion could alter the bacterial co-occurrence pattern, especially the abundant subcommunity, corresponding to a greater variation in bacterial composition of abundant taxa.

In the light of the null model analysis, the results indicated that both deterministic and stochastic assembly regulated assembly of the bacterial community (Supplementary Figures 8, 9). The NST values were used to determine a stochastic (>50%) or deterministic (<50%) assembly (Ning et al., 2019; Zhou et al., 2020). Here, a stochastic assembly was observed for the rare taxa (NST > 50%) when compared with the abundant taxa (NST < 50%) (Figure 7), which agrees with the results of previous studies (Jiao et al., 2017; Du et al., 2020). We hypothesized that due to their relatively small population size, the demographic stochasticity and dispersal had a significant impact on the rare taxa (Jousset et al., 2017). Moreover, we observed a gradual decrease in the impact of the stochastic processes on the abundant taxa post-microbial invasion (Figure 7), showing the role of homogenizing selection or variable selection increased along with the cumulative effects of O157:H7 invasion. To fully understand the bacterial community assembly mechanisms, future research should consider the sampling scale effects (spatial extent and time scale), more important explanatory deterministic factors (e.g., unmeasured environmental factors and species interactions), and other possible stochastic factors.

## Conclusion

Microbial invasions are a threat to ecosystems, and the response of native microbial communities revealed the ecological risk of the microbial invader. Divergent responses of abundant and rare sediment taxa to invading *E. coli* O157:H7 were observed in this study. The rare taxa were found to be more stable to *E. coli* O157:H7 invasion. Alternatively, there might have been certain changes without an accompanying change in community membership which could not be detected by 16S rRNA genes sequencing. Omics technology could be adopted to reveal more detailed changes caused by *E. coli* O157:H7 invasion. In addition, functional analyses are needed to understand the functional impact of microbial invasion on sediment native microbiota in detail.

## Data Availability Statement

The datasets presented in this study can be found in online repositories. The names of the repository/repositories and accession number(s) can be found below: https://www.ncbi.nlm.nih.gov/, PRJNA669260.

## Author Contributions

NZ: writing, formal analysis, and investigation. CL and XL: investigation and formal analysis. ZY: formal analysis, supervision, writing—review and editing, and investigation. DZ: supervision and writing—review and editing. SD and HZ: formal analysis.

## Conflict of Interest

The authors declare that the research was conducted in the absence of any commercial or financial relationships that could be construed as a potential conflict of interest.

## Footnotes

1 https://qiime2.org/

2 http://www.r-project.org
