# Supplementary material for: Divergent Temporal Response of Abundant and Rare Bacterial Communities to Transient Escherichia coli O157:H7 Invasion
Source: Front Microbiol. 2021 Jun 7;12:665380. doi: 10.3389/fmicb.2021.665380 (PMC8215281; doi:10.3389/fmicb.2021.665380)
Supplement: Supplementary file 1 [file Data_Sheet_1.docx]

**Supplementary Table and Figure legends**

**Table S1** Multivariate tests for variation in Shannon index among the treatments.

**Figure S1** Map showing the sampling stations. Red point showed the sampling sites in the estuarine marsh in south Hangzhou Bay.

**Figure S2** Variation in α-diversity of the bacterial subcommunity across the sampling day. (A) Abundant taxa of the B sediment (B) Rare taxa of the B sediment (C) Abundant taxa of the B sediment (D) Rare taxa of the P sediment. CON, the control group; INV, the invasion group.

**Figure S3** Taxonomic distribution of the 12 most abundant phyla (relative abundance above 1%) of the bacterial subcommunities in abundance and rarity in the sediments. (A) B sediment (B) P sediment. * represents the level of significance examined by the nonparametric Mann-Whitney U test (*p* < 0.05). Abundant: abundant bacterial subcommunity. Rare: rare bacterial subcommunity.

**Figure S4** Temporal variation in bacterial composition at the phyla level (relative abundance above 1%). (A) Abundant taxa of the B sediment (B) Abundant taxa of the P sediment (C) Rare taxa of the B sediment (D) Rare taxa of the P sediment. CON, the control group; INV, the invasion group.

**Figure S5** Principal-coordinate analysis (PCoA) of the sub bacterial communities based on the Weighted Unifrac distance. (A) Abundant bacterial subcommunity of the B sediment (B) Abundant bacterial subcommunity of the P sediment (C) Rare bacterial subcommunity of the B sediment (D) Rare bacterial subcommunity of the P sediment. CON, the control group; INV, the invasion group.

**Figure S6** The influence of species turnover and nestedness-resultant richness towards the total β-diversity of the bacterial community of the B (A) and P (B) sediments.

**Figure S7** Co-occurrence patterns of all bacterial community. The profiles of co-occurrence links among dominant taxa. Connections are colored based on the most dominant taxon (relative abundance above 2%). CON, the control group; INV, the invasion group. (A) B sediment of the CON group (B) B sediment of the INV group (C) P sediment of the CON group (D) P sediment of the INV group.

**Figure S8** The neutral community model (NCM) fit of community assembly to structure the bacterial community. (A) the whole community of B sediment of the CON group (B) three subcommunities of B sediment of the CON group (C) the whole community of B sediment of the INV group (D) three subcommunities of B sediment of the CON group. The solid red lines indicate 95%CI above the model prediction, and the solid blue line is the 95%CI below the model prediction. Different colors are used to indicate ASVs that occur in variable frequency than predicted by the NCM. *m* stands for the metacommunity migration rate, R refers to the fit to neutral model. Numbers in the bar plot refer to the ASV counts.

**Figure S9** The neutral community model (NCM) fit of community assembly to structure the bacterial community. (A) the whole community of P sediment of the CON group (B) three subcommunities of P sediment of the CON group (C) the whole community of P sediment of the INV group (D) three subcommunities of P sediment of the CON group. The solid red lines indicate 95%CI above the model prediction, and the solid blue line is the 95%CI below the model prediction. Different colors are used to indicate ASVs that occur in variable frequency than predicted by the NCM. *m* stands for the metacommunity migration rate, R refers to the fit to neutral model. Numbers in the bar plot refer to the ASV counts.

**Table S1** Multivariate tests for variation in Shannon index among the treatments.

| Group | | Shannon | | | | | |
| --- | --- | --- | --- | --- | --- | --- | --- |
|  |  | WT | | AT | | RT | |
|  |  | F | P | F | P | F | P |
| B | INV | 0.362 | 0.553 | 7.154 | 0.013 | 0.052 | 0.821 |
|  | TIME | 3.206 | 0.032 | 2.040 | 0.124 | 3.031 | 0.039 |
|  | INV*TIME | 0.071 | 0.975 | 0.288 | 0.834 | 0.126 | 0.943 |
| P | INV | 1.910 | 0.177 | 0.141 | 0.710 | 0.630 | 0.433 |
|  | TIME | 0.963 | 0.458 | 1.508 | 0.220 | 0.485 | 0.784 |
|  | INV*TIME | 0.075 | 0.989 | 0.399 | 0.807 | 0.295 | 0.878 |

WT: Whole; AT: Abundant taxa; RT: Rare taxa.


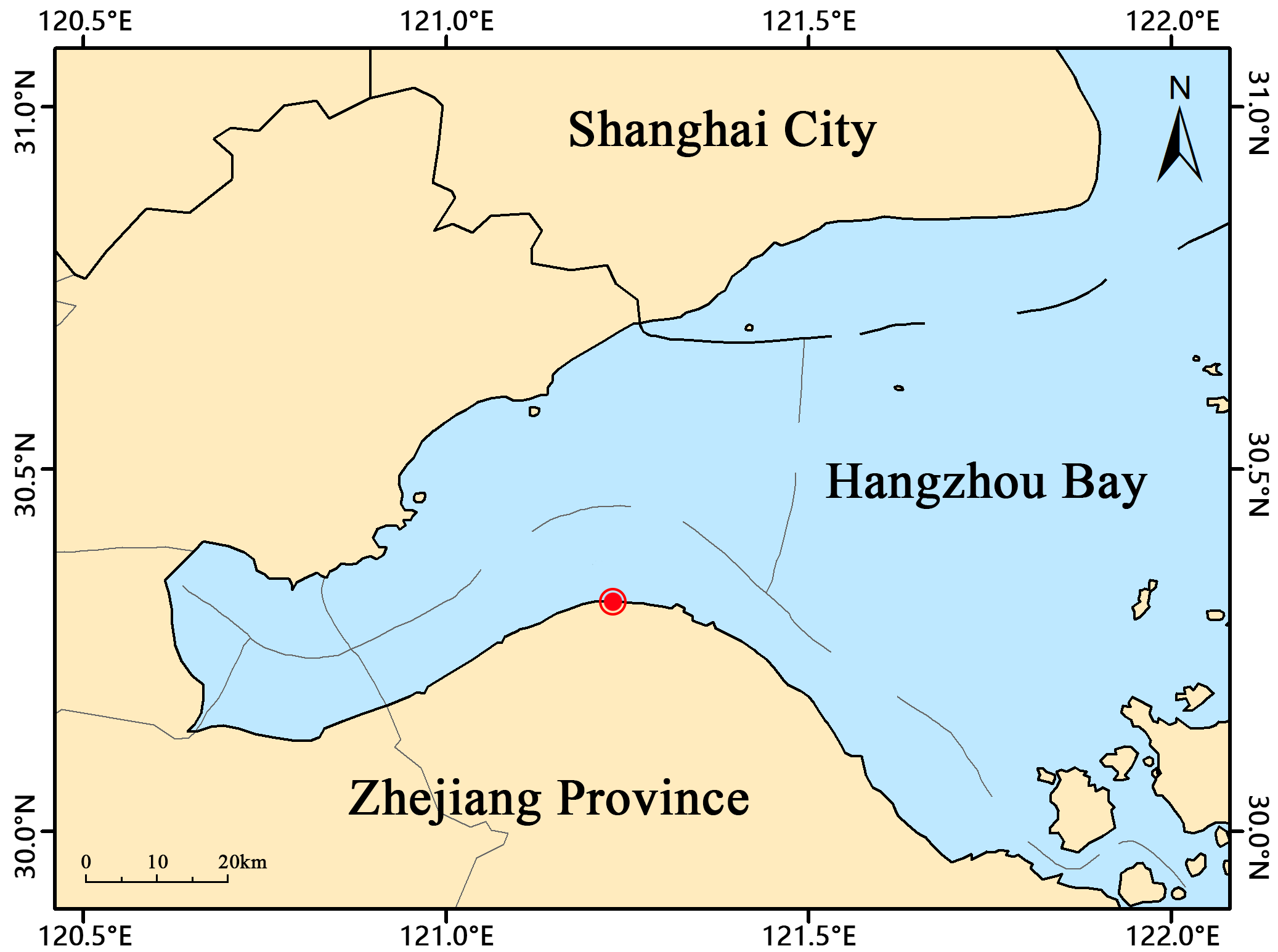


**Figure S1** Map showing the sampling stations. Red point showed the sampling sites in the estuarine marsh in south Hangzhou Bay.


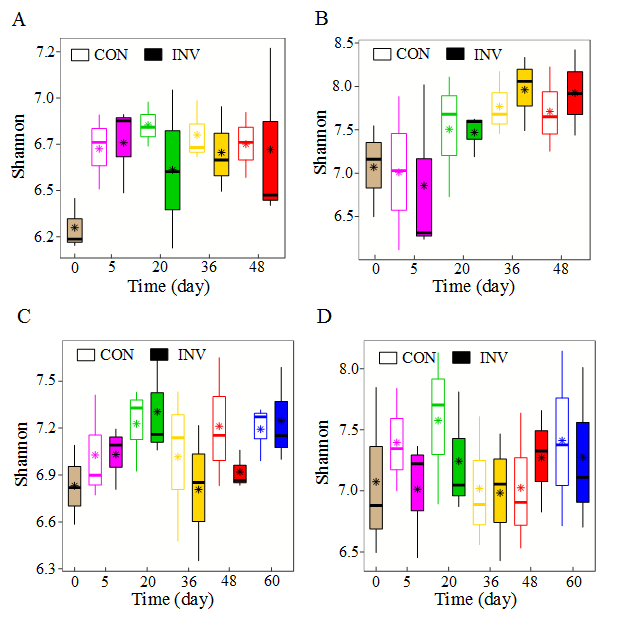
**Figure S2** Variation in α-diversity of the bacterial subcommunity across the sampling day. (A) Abundant taxa of the B sediment (B) Rare taxa of the B sediment (C) Abundant taxa of the B sediment (D) Rare taxa of the P sediment. CON, the control group; INV, the invasion group.


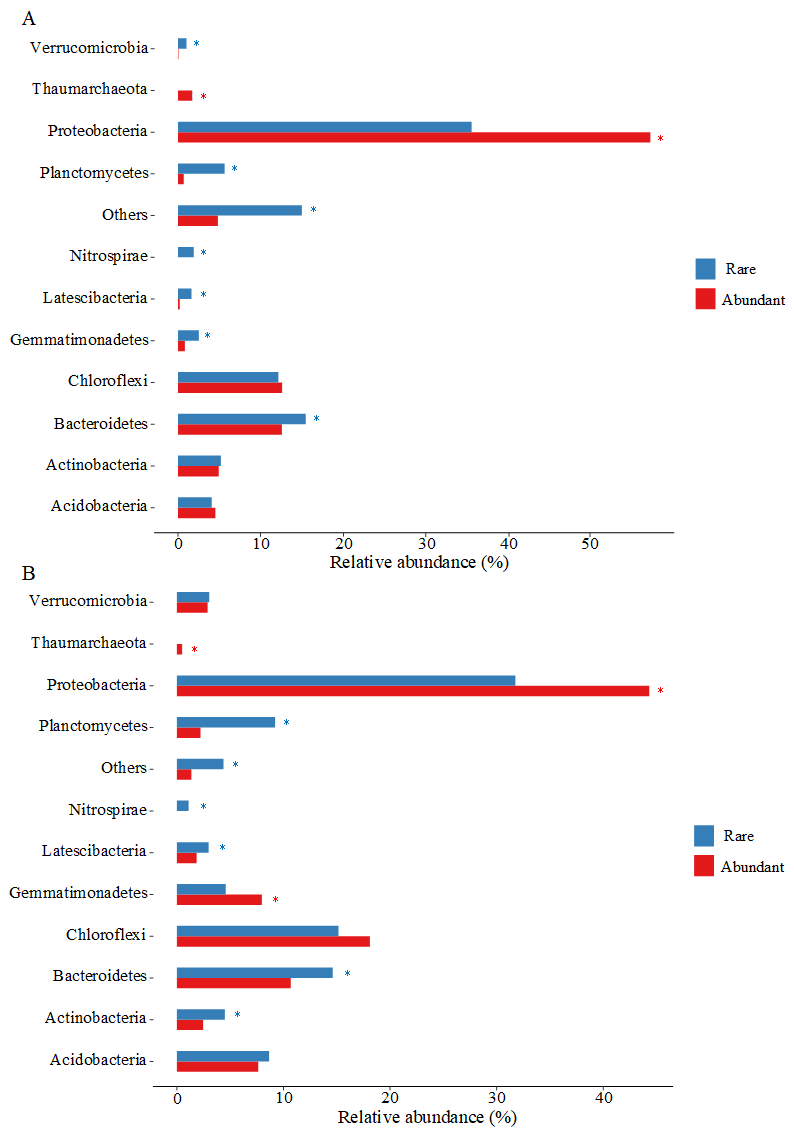


**Figure S3** Taxonomic distribution of the 12 most abundant phyla (relative abundance above 1%) of the bacterial subcommunities in abundance and rarity in the sediments. (A) B sediment (B) P sediment. * represents the level of significance examined by the nonparametric Mann-Whitney U test (*p* < 0.05). Abundant: abundant bacterial subcommunity. Rare: rare bacterial subcommunity.


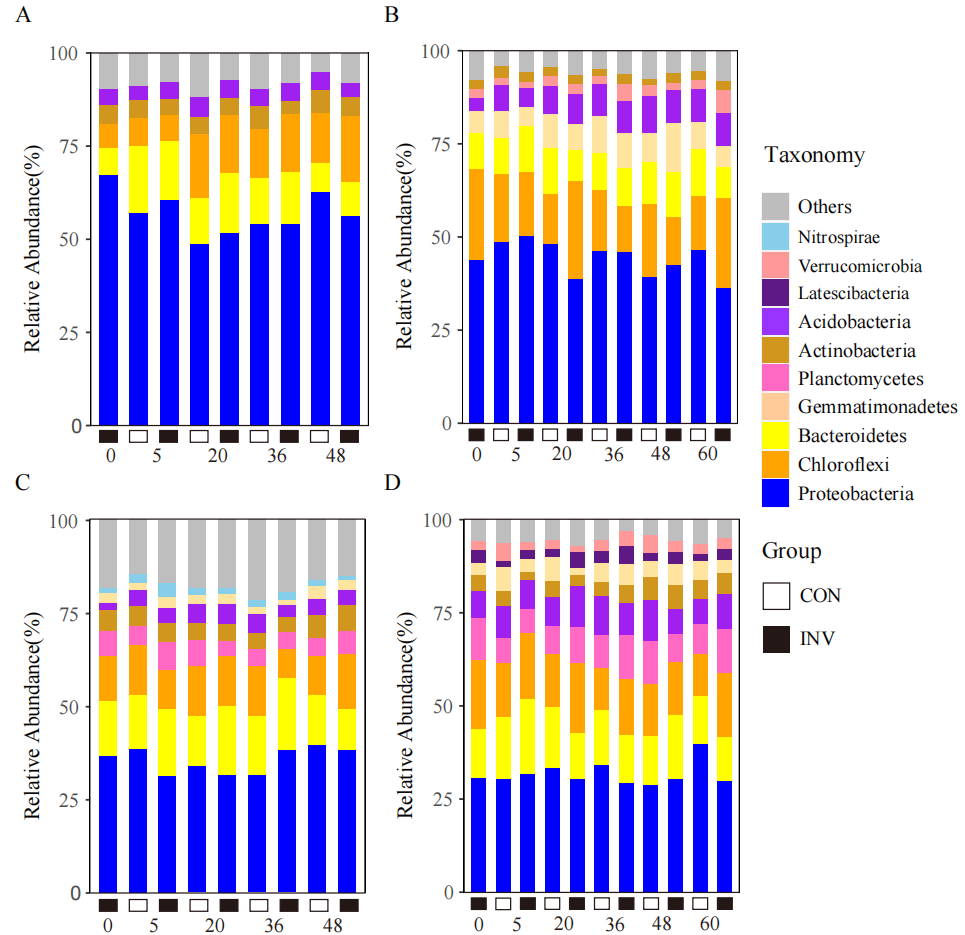


**Figure S4** Temporal variation in bacterial composition at the phyla level (relative abundance above 1%). (A) Abundant taxa of the B sediment (B) Abundant taxa of the P sediment (C) Rare taxa of the B sediment (D) Rare taxa of the P sediment. CON, the control group; INV, the invasion group.


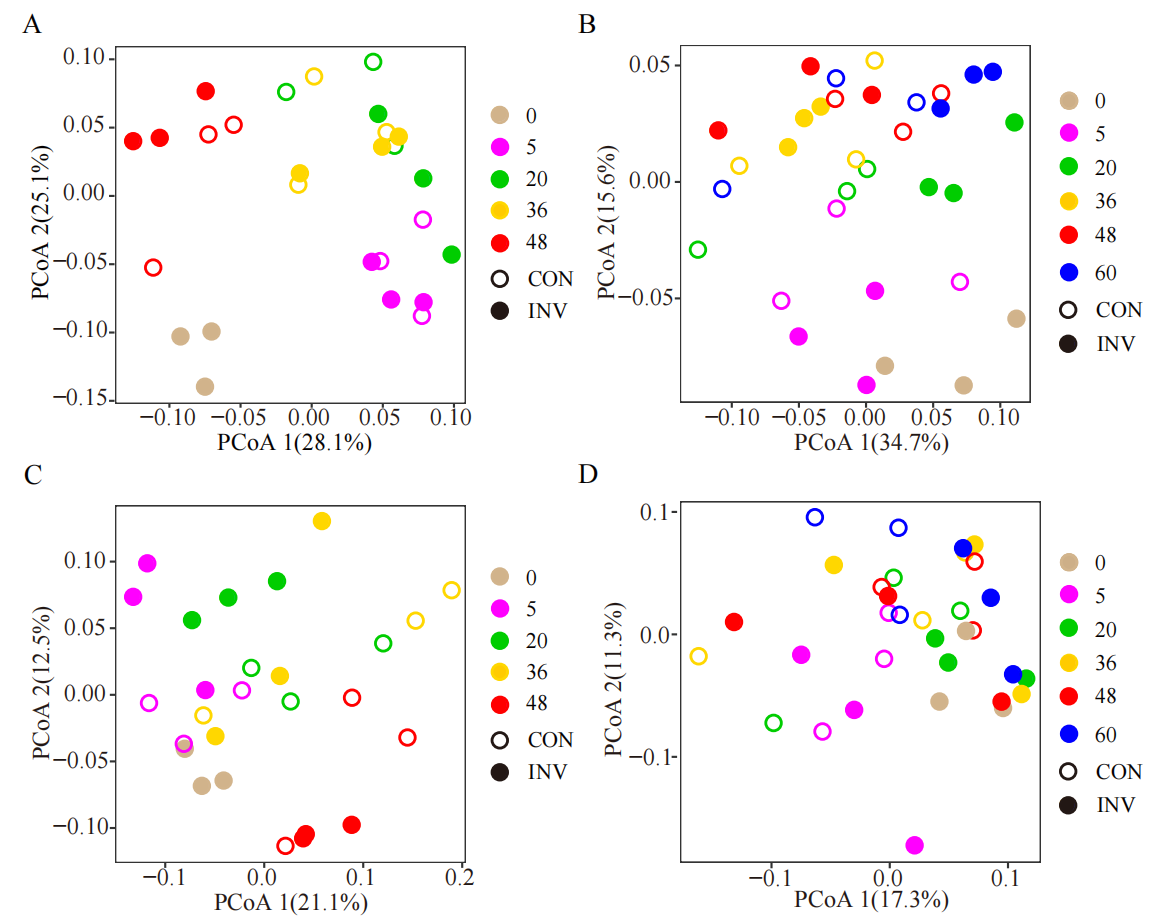


**Figure S5**: Principal-coordinate analysis (PCoA) of the sub bacterial communities based on the Weighted Unifrac distance. (A) Abundant bacterial subcommunity of the B sediment (B) Abundant bacterial subcommunity of the P sediment (C) Rare bacterial subcommunity of the B sediment (D) Rare bacterial subcommunity of the P sediment. CON, the control group; INV, the invasion group.


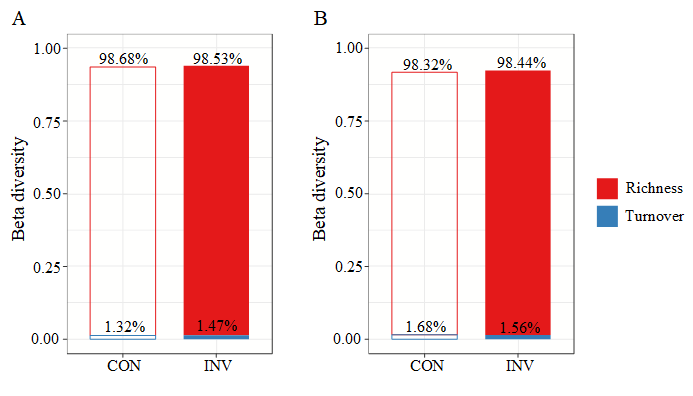


**Figure S6**: The influence of species turnover and nestedness-resultant richness towards the total β-diversity of the bacterial community of the B (A) and P (B) sediments.


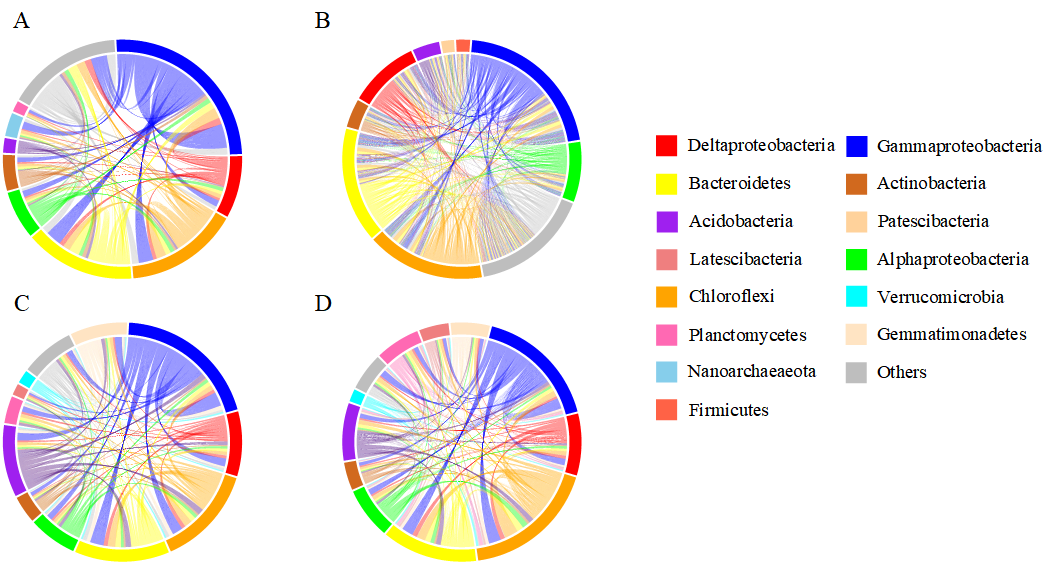


**Figure S7** Co-occurrence patterns of all bacterial community. The profiles of co-occurrence links among dominant taxa. Connections are colored based on the most dominant taxon (relative abundance above 2%). CON, the control group; INV, the invasion group. (A) B sediment of the CON group (B) B sediment of the INV group (C) P sediment of the CON group (D) P sediment of the INV group.


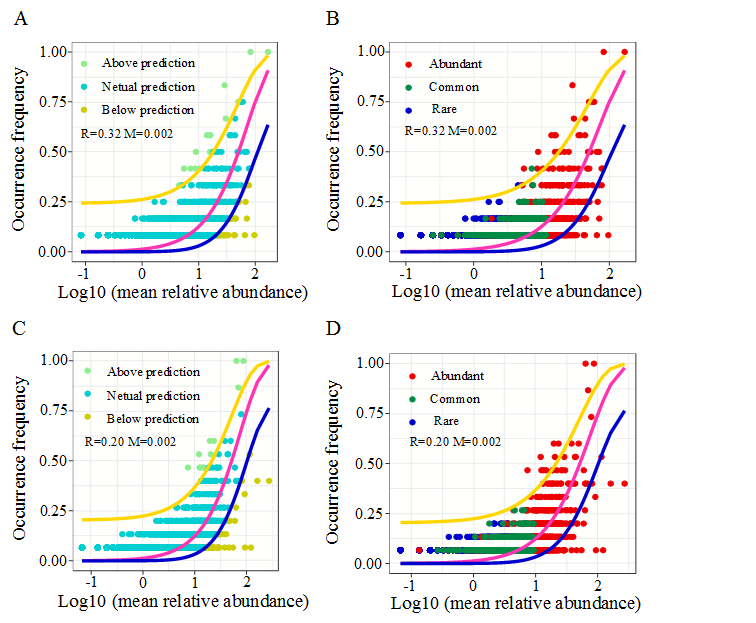


**Figure S8** The neutral community model (NCM) fit of community assembly to structure the bacterial community. (A) the whole community of B sediment of the CON group (B) three subcommunities of B sediment of the CON group (C) the whole community of B sediment of the INV group (D) three subcommunities of B sediment of the CON group. The solid red lines indicate 95%CI above the model prediction, and the solid blue line is the 95%CI below the model prediction. Different colors are used to indicate ASVs that occur in variable frequency than predicted by the NCM. *m* stands for the metacommunity migration rate, R refers to the fit to neutral model. Numbers in the bar plot refer to the ASV counts.


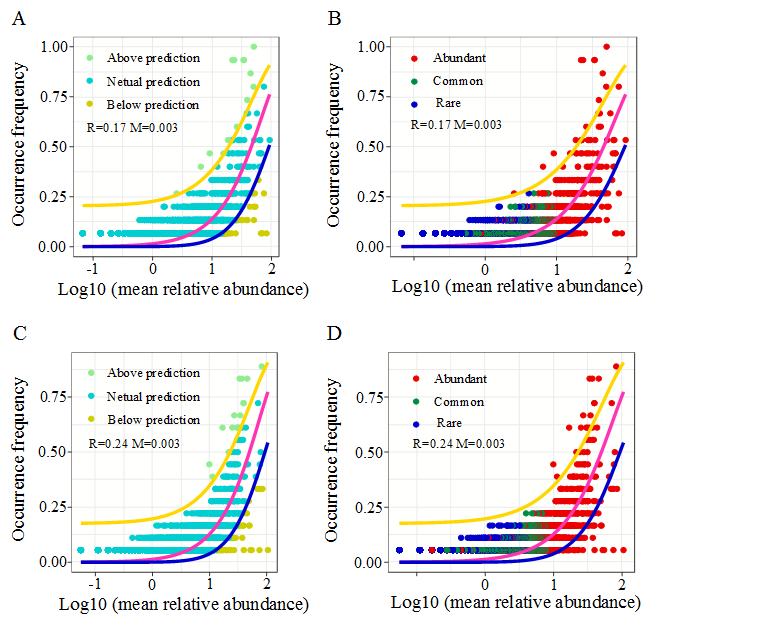


**Figure S9** The neutral community model (NCM) fit of community assembly to structure the bacterial community. (A) the whole community of P sediment of the CON group (B) three subcommunities of P sediment of the CON group (C) the whole community of P sediment of the INV group (D) three subcommunities of P sediment of the CON group. The solid red lines indicate 95%CI above the model prediction, and the solid blue line is the 95%CI below the model prediction. Different colors are used to indicate ASVs that occur in variable frequency than predicted by the NCM. *m* stands for the metacommunity migration rate, R refers to the fit to neutral model. Numbers in the bar plot refer to the ASV counts.
